# The psychological impact of using 3D printing and imaging technology for patient education: a scoping review

**DOI:** 10.1186/s41205-026-00327-9

**Published:** 2026-05-15

**Authors:** Kayleigh Maxwell, Steve Leung, Gozde Ozakinci

**Affiliations:** 1 Department of Urology, Western General Hospital, Edinburgh, EH4 2XU UK; 2 Division of Psychology, Faculty of Natural Sciences, University of Stirling, Stirling, FK9 4AL UK

**Keywords:** 3D technology, Patient outcomes, Anxiety, Patient-health care professional communication, Patient education

## Abstract

**Background:**

The way in which patient education is delivered during clinical consultations can have an impact on cognitive and emotional outcomes in patients. 3D printing and imaging can be used in patient education to improve understanding of the information and satisfaction with care. This scoping review sought to explore the psychological impact of using 3D models in patient education.

**Methods:**

Searches were conducted in PsycINFO, PsycARTICLES, PubMed, Medline and CINAHL. Levac et al.’s enhanced version of Arksey & O’Malley’s methodological framework for conducting scoping reviews, and the PRISMA-ScR, were used to guide the screening and identification of relevant studies. Studies were included if they investigated the effect of using 3D models in patient education and explored psychological outcomes. Both quantitative and qualitative research were included.

**Results:**

Eleven studies were included in the review, including 2 qualitative studies. 3D models were most often used in educational consultations preceding a surgical procedure (*n* = 9). Psychological outcomes assessed were anxiety, quality of life, distress relief, and decisional conflict. The results were mixed, showing that using 3D models can have a positive as well as negative effect on psychological outcomes such as fear and disempowerment.

**Conclusions:**

Using 3D models in patient education has the potential to improve patient anxiety and other psychological outcomes. However, more research is required to identify which patients and types of consultations 3D models are most useful for. For example, appointments involving important decision-making may benefit from the inclusion of 3D models. It is also essential to consider the communicative approach of the healthcare professional in the delivery of patient education with 3D models, as this factor is key to the outcomes of shared decision-making.

## Introduction

Receiving information from a healthcare professional, whether relating to a newly diagnosed condition or plans for a treatment like surgery, can evoke a significant amount of anxiety in patients. Patient understanding of the presented information is not always prioritised and consultations can leave patients feeling dissatisfied and with questions left unanswered [1]. Anxiety levels during clinical consultations can be particularly elevated amongst populations facing a life-threatening illness such as cancer, with an estimated 10–50% of cancer patients experiencing high anxiety in these consultations [2]. Effective communication around diagnosis and treatment planning is essential for reducing patient anxiety and improving patient wellbeing.

Patient education includes the provision of information about a diagnosis or about a surgical procedure. The way in which such education is delivered can affect psychological outcomes [3]. Education may be delivered in a written format, or a digital format using images or scans and may be conducted via a consultation with a health care professional face to face or using telephone or telemedicine [3, 4]. The provision of key information relating to the patient’s health and treatment pathway, delivered in a way which prioritises patient understanding and satisfaction, has the potential to improve cognitive and affective psychological outcomes such as anxiety, depression, and negative thinking [5, 6].

Evidence shows that patient education using CT (Computed Tomography), or MRI (Magnetic Resonance Imaging) images can be most effective for giving an accurate representation of the anatomy and physiology of a medical issue [7]. However, this involves the presentation of complex medical information which patients do not always find easy to understand [8]. Three-dimensional (3D) models offer an alternative method for presenting such medical information to patients. This approach involves the creation of virtual or physical 3D objects from two-dimensional images of anatomical structures [9], which has the potential to aid patient understanding, providing clearer and often personalised representations of a medical issue [10].

There is a wealth of literature demonstrating how 3D models may be used to increase patient understanding and satisfaction, particularly in research on urological conditions affecting the kidneys, bladder, or prostate [11–16]. Much of the literature on 3D modelling in healthcare also focuses on its use in training health professionals and surgical planning [17–20]. Given that psychological outcomes such as anxiety can be significantly affected by the quality of medical consultations, it is surprising to find that, while 3D models have been trialled as a patient education tool with a variety of patient groups, there is a scarcity of research investigating the effect of using 3D models on psychological outcomes.

As the literature on this topic is sparse, a scoping review was deemed the most appropriate methodology, as it allowed for a wide-ranging approach to address the aim of this review, which was to identify relevant studies which explore the effect of using 3D models in patient education on psychological outcomes.

## Methods

Levac et al.’s [21] enhanced methodology for using Arksey & O’Malley’s [22] framework was employed for this scoping review. This methodology offers recommendations for enhancing the original five stages of the framework: (1) identifying the research question, (2) identifying relevant studies, (3) study selection, (4) charting the data, and (5) collating, summarising, and reporting the results. The Preferred Reporting Items for Systematic reviews and Meta-Analyses extension for Scoping Reviews (PRISMA-ScR) Checklist [23] was also consulted in the process of clarifying the research question and identifying relevant studies.

### Stage one: Identifying the research question

In order to clarify the focus of the review and build an effective search strategy, the research question considers a predetermined concept, target population and health outcome, that is, 3D models for patient education, patient populations, and psychological outcomes. Studies were sought which utilised 3D technology in the form of printed or physical 3D models, digital 3D models (computer-generated images shown on a screen), or Virtual Reality (VR) models. Preliminary scoping of the literature showed that 3D models have been investigated in a range of patient populations, and therefore we did not choose to focus on any specific patient or disease group. We also sought to include studies which had investigated any psychological outcomes, as the literature on this topic is yet to be summarised in a review. PRISMA-ScR was used to further articulate the research objectives, inclusion criteria and search terms. The following search strategy was used (this version formatted for use in the PubMed database):

(surgery OR surgical OR operati*) AND ((3D OR 3-D OR “3 dimension*” OR three-dimension* OR “three dimension*”) AND (imaging OR modelling OR printing)) AND (“patient education” OR “surgical education” OR “patient counselling” OR “patient teaching” OR “patient learning” OR “patient information” OR “health knowledge” OR “health literacy”).

### Stage two: Identifying relevant studies

The research team worked together to screen the identified studies for relevant literature. At first, the search strategy included keywords “anxiety” and “depression”, but the number of studies identified was extremely low, so an iteration was made to exclude specific psychological keywords to avoid missing relevant studies. Both quantitative and qualitative studies were included to capture the breadth of studies to answer the research question. The following databases were used to search for literature: PsycINFO, PsycARTICLES, PubMed, Medline and Cinahl. Searches were conducted in August 2024 and were not limited by publication date. Identified studies were then imported into the Rayyan software (available at https://www.rayyan.ai/) and duplicates were removed.

### Stage three: Study selection

**Fig. 1:**
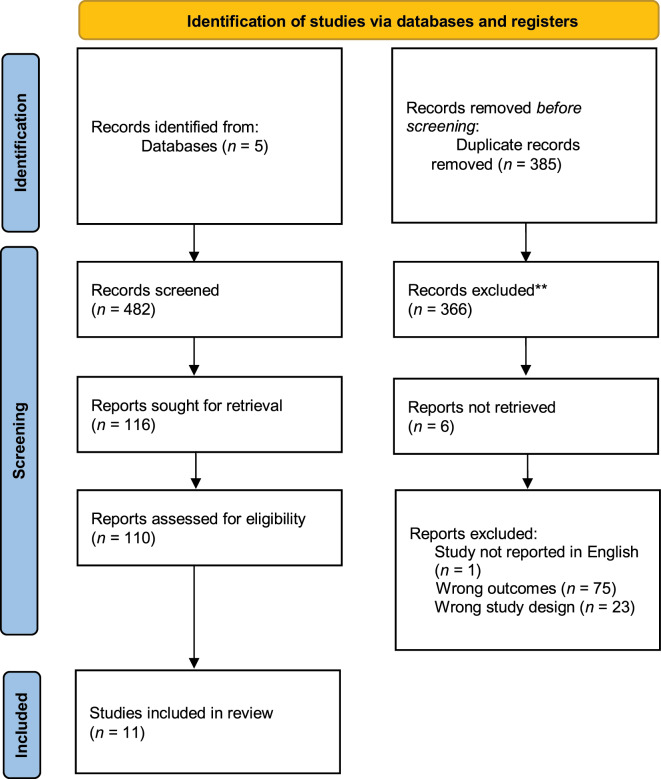
PRISMA flowchart of studies

Two of the research team (KM and SL) worked together in Rayyan to screen the identified studies and make decisions on which studies were relevant. Studies were included if they reported on the use of either physical 3D models, digital 3D models, or VR models, if these models were used for patient education, and at least one psychological outcome was measured. Articles were excluded if they did not provide primary evidence (e.g., protocols or reviews), they only measured patient education and/or patient satisfaction but no other psychological variable, or they were reported in languages other than English. The titles and abstracts of 482 studies were screened initially and 366 were excluded as they did not meet the inclusion criteria. The full texts of the remaining 116 studies were then assessed against inclusion criteria, with a total of 11 studies being included in the review. The second reviewer (SL) screened 25% of the total number of studies at both the title and abstract stage and the full-text stage to ensure that decisions were made in line with the overall purpose of the review. Reference lists of key papers were also screened, and any relevant studies added. The team met regularly to discuss and resolve any queries. A full outline of the identification process can be seen in Fig. 1.

### Stage four: Charting the data

The first author (KM) created a chart including the following information “Authors; Year of publication; Study design; Type of surgery or condition; Sample size; Participant demographics; Country of study; Description of 3D models used; Psychological outcomes measured and whether the results were positive or negative; Relevant qualitative data (if reported).”

SL and GO reviewed the data chart to ensure that the data extraction process was consistent with the objectives of the review. The final list of psychological outcomes was as follows: anxiety, quality of life, self-efficacy, distress, and decisional conflict. Extracted qualitative data included primary data (participant quotes) and secondary data (author’s interpretations).

### Stage five: Collating, summarising, and reporting the results

Quantitative results were collated and summarised in a numerical summary table and psychological constructs were extracted from the qualitative data and presented in a table. A narrative descriptive summary was written to explain what the collated findings mean in relation to the research question and what the implications of this review are for future research and clinical practice.

## Results

### Study characteristics

Eleven studies were identified as being relevant for the review. The included studies were published between 2016 and 2023 and primarily conducted in the USA [24–29], but also Germany [30], China [31], the UK [32], Korea [33], and the Netherlands [34]. The total sample across studies was 405 and included patients undergoing surgeries [24, 25, 27–30, 32–34], patients diagnosed with benign paroxysmal positional vertigo [26], and patients diagnosed with urological issues [31]. Five studies used randomised controlled trials (RCTs) [25, 26, 28, 30, 31], and there were also two qualitative studies [32, 34], one case report (with pre- and post-intervention measurement) [33], one feasibility study (one group receiving personalised 3D model with pre-post intervention measures) [29], one pilot study (with pre-post intervention measures) [24], and one prospective pilot study (with post-intervention measurement only with no control group) [27]. McDonald and Shirk [27] investigated the use of 3D models in both telehealth and in-person consultations. In the case of qualitative studies, only patient related quotes and authors’ interpretations have been analysed. See Table 1 for full details on study characteristic.

**Table 1:** Characteristics of the included studies

Author(s)/year and country	Patient population Sample size (*N*) Age (Mean or median (SD or range or %) Sex *n* (%)	Study design and 3D model type	Measures if applicable and key findings	Limitation
Benson et al. (2022) – USA [24]	Patients undergoing surgical resection for lung cancer Intervention group (N = = 22): Age: 66.5 (62–74) 7 males (31.8) 15 females (68.2) Control group (*N* = 26): Age: 69 (58–72) 10 males (38.5) 16 females (61.5)	Pilot pre-post intervention study (2 groups and no control group). Groups: Multimedia intervention (ME) or standard preoperative education Digital 3D models (had generic and personalised elements) as part of a multimedia educational platform that include video tutorials and patient radiology images). Participants also received all the annotated 3D models and personalised scan images as a printed copy for their records.	Primary outcomes: Lung cancer knowledge, quality of life (measured by FACT-L.), patient satisfaction. Health literacy (eHealth literacy) was also measured. No difference in quality of life between groups. Additionally, no difference in knowledge scores and nor eHealth literacy. ME group had higher satisfaction compared to standard education.	Could be under-powered as it was a pilot study with no power calculations.
Biro et al. (2019) – USA [25]	Patients undergoing Mohs micrographic surgery (MMS) for removal of non-melanoma skin cancer Intervention group (*N* = 42): Age: 67.83 (11.34) Sex: 24 males (57.14) 18 females (42.86) Control group (*N* = 40): Age 67.83 (12.89) Sex: 25 males (62.5) 15 females (37.5)	RCT (2 groups, pre and post measurements). Groups: Mohs micrographic surgery (MMS) model plus standardised education (SE) or SE alone. Printed generic 3D model The SE group received verbal counselling from a standardized script about the MMS procedure (5 min) and the MMS group received SE as well generic printed 3D model of the skin and tumour.	Primary outcome: Anxiety measured by STAI and VAS. Secondary outcomes: patient understanding and satisfaction. Both groups had reduced anxiety (VAS and STAI). Reduction in anxiety was significantly greater for the intervention group. The difference in anxiety reduction between the two groups approached statistical significance (*P* = .052) on the VAS scale and there was no difference on STAI. Mixed findings with regard to impact of 3D printed models on anxiety.	Power analysis has been conducted but could be under-powered (reached 91.1% intended sample size). Baseline anxiety was not as high as anticipated.
Fontenot et al. (2023) - USA [26]	Patients with benign paroxysmal positional vertigo Experimental group (*N* = 8) Age: 49.4 (15.0) 1 male (12.5) 7 females (87.5) Control group (*N* = 8) Age: 43.3 (12.9) 3 males (37.5) 5 females (62.5)	Single centre RCT (2 groups, pre- and post-intervention measurements). Groups: Experimental (3D printed generic vestibular model and standardised verbal education) or control (standardised verbal education)	Patient understanding of illness aetiology; comfort level with symptom prevention; anxiety related to knowledge (measured by “*Does your current level of knowledge regarding your dizziness cause you to feel any stress or anxiety?”).* Authors state that the mean decrease in anxiety related to symptoms was 1 in 3D model and 0.13 in control group which was significant.	Small sample size (no effect size reported). Anxiety is measured with one item and in relation to knowledge about condition developed for the purpose of study therefore not possible to compare findings across studies. Confusion in article regarding what anxiety item measured (knowledge or symptoms).
Grab et al. (2023) – Germany [30]	Patients undergoing cardiac surgery *N* = 99 Age: Printed 3D models (*N* = 34): 66.15 (10.06) VR models (*N* = 31): 65.97 (8.02) Control (*N* = 34) 62.94 (13.94) Sex: Printed 3D models: 29 males (85.29) 5 females (14.71) VR models: 26 males (83.87) 5 females (16.13) Control: 32 males (94.12) 2 females (5.88)	RCT (3 groups, 3 assessments at pre- and post- education and at least 1 week after surgery or at discharge). Groups: standardised paper-based; generic 3D printed models for different surgical options; generic virtual reality (VR) for different surgical options).	Primary outcome: Anxiety measured by Visual Analog Scale (VAS, 1–10) and State-Trait-Anxiety-Inventory (STAI) – both state and anxiety scores. STAI-Trait was measured 1 week after surgery) Secondary outcomes: procedural understanding and patient satisfaction. The only statistically significant reduction in anxiety observed VR model group which showed significant reductions in VAS score (not in STAI), Control group and the 3D-printed model group only showed slight, non-significant reductions in VAS anxiety. STAI scores were slightly reduced for the control group and slightly increased for the 3D-printed group, but neither change was significant. No significant differences in the Trait-Anxiety-Score in groups when measured at least one week after surgery.	
Hu et al. (2016) – China [31]	Patients with urological issues *N* = 240 Experimental group (*N* = 120) Age: 18–32 years old − 20 (16.67%), 33–47 years old − 35 (29.17%), 48–62 years old − 39 (32.5%), 63 years old − 26 (21.67%) Sex: 80 males (66.7) 40 females (33.3) Control group (*N* = 120) 18–32 years old − 14 (11.67%), 33–47 years old − 33 (27.5%), 48–62 years old − 36 (30%), 63 years old – 37 (30.83%)	RCT (2 groups, single time point measurement- only immediately post-intervention assessment). Groups: 3D generic printed model or ‘pictures-based communication’ using the model-design pictures explaining testing, cause and diagnosis, treatment and effects, side effects and risks, and prognosis.	Medical Interview Satisfaction Scale (MISS): overall satisfaction; distress relief; communication comfort; rapport; compliance intent. 3D generic print group had significantly higher patient satisfaction, promoting patient distress, improving communication comfort, rapport, and complication intent compared to control group.	No pre-intervention assessment
McDonald & Shirk (2023) – USA [27]	Patients undergoing renal mass treatment *N* = 47 (*N* = 35 telehealth and *N* = 12 in-person visits) No information on age or sex	Prospective, single-arm cohort study (2 groups, single time point measurement post-consultation). Groups: telehealth or in-person Digital personalised 3D models showing their kidney, renal mass, and key adjacent structures were included in patient consultation visits as an additional tool for patient counselling.	Increased understanding of the condition and treatment options; reduction in anxiety (“*The 3D model helped reduce my concern or anxiety about my condition or treatment.”;* influence on treatment and physician choice and whether the addition of 3D model made the virtual consultation as effective as an in-person visit (1–5 Likert scale, high scores indicating higher understanding/higher reduction in anxiety). Increased understanding of treatment options and decreased anxiety of the condition were reported. Having 3D models in telehealth visits were felt as making the visit as effective as an in-person visit. Free-text themes showed better understanding of disease; more comfort/confidence in treatment; better visualisation of disease. No differences in outcomes between telehealth and in-person groups.	No pre-consultation assessment. No control group
Phelps et al. (2020) – UK [32]	Patients with hip injury (femoroacetabular impingement) *N* = 14 (patients, 12 of whom seen 3D images (7 of whom were shown other images as well) and 2 only 2D images; *N* = 4 health care professionals; *N* = 31 lay representatives in 6 focus groups, 9 of whom are from community patient groups shown a variety of images relating to hip injuries and cancer, and colonoscopy) Patient participants: Age: 20–29 years old − 4 (28.6%), 30–39 years old − 2 (14.3%), 40–51 years old − 8 (57.1%) Sex: 9 males (64.3) 5 females (35.7)	Qualitative (only data from patients are used for this review) Digital personalised 3D models (in addition to X-rays, MRI images, 2D CT images)	Themes from patients included: The truthful image (as evidence and trust in image). ‘Overuse of imaging’ reported in focus group (not clear if data are from patients) The empowering image (helping ‘make sense’ and communicate to family, friends, and employer; making decisions, and helping ‘moving forward’. The unhelpful image: causing distress or disempowering	Focus group participants included community patient groups but not possible to disaggregate their data.
Rhu et al. (2022) – Korea [33]	A patient with liver cancer *N* = 1 Age: 66 1 female	Case report (pre and post measurements) VR model of patient’s liver	Anxiety was measured using State-Trait Anxiety Inventory-X (STAI-X)-1 and STAI-X-2. Knowledge of liver surgery and patient-specific information. Both anxiety scores increased following VR session. Knowledge improved following VR session.	Case study. The conditions before and after the VR where measurements are taken are not consistent (e.g., presence of family member or not).
Sander et al. (2017) – USA [28]	Patients undergoing sinus or nasal surgery *N* = 100 No information on sex or age (all over the age of 18)	RCT (2 groups, post-intervention measurement). Groups: Intervention (generic 3D printed model of the nasal sinus anatomy of a patient + standard information) or standard information (information on anatomy, disease, surgical options using 2D charts and verbal explanation)	Patient understanding of anatomy, disease state, and surgical options; anxiety reduction (1 item “*Did the explanation ease your anxiety?”* yes; no; n/a). Statistically significant difference between groups in terms of understanding items. No statistically significant difference between groups in terms of easing anxiety but agreement with the item was high in both control (91.4%) and 3D model group (97.5%).	No pre-intervention assessment Averages reached ceiling score making it difficult to ascertain the true effect of 3D models. Not clear whether it was powered adequately.
Santiago et al. (2021) – USA [29]	Women undergoing surgery for breast cancer *N* = 25 Age: 48.8 (range 28 − 72)	Feasibility (acceptability) study (1 group, pre and post measurements) Personalised 3D printed model	Treatment-related decisional conflict (Decisional Conflict Scale) Acceptability of 3D using Ottawa acceptability questionnaire Significant reduction in overall decisional conflict and 4 out of 5 subscales – no difference in support subscale. Acceptability of personalised 3D printed models were high but emotional response was more variable	Feasibility study so no control group and no pre-consultation assessment.
van de Belt et al. (2018) – Netherlands [34]	Adult patients with glioma who underwent fMRI (*N* = 11) Age: 43.2 (11) 6 males (55) 5 females (45)	Qualitative interviews Personalised 3D printed model	18 facilitators and 6 barriers were identified: Coping and acceptance of the condition could be improved. Seeing the model might be an emotional experience especially in the early part of the treatment. Anger and fear ere mentioned. Seeing tumour as part of oneself, instead of something frightening. Increased ability to communicate with neurosurgeon and family and friends are mentioned.	Not many patient quotes have been provided.

### Summary of studies’ 3D features

The studies used different types of 3D models. Seven studies used printed 3D models [25, 26, 28–31, 34], four used digital 3D models [24, 27, 32], and two used VR models [30, 33]. The models were used for patient education, either to aid education about an upcoming surgery, or education about a health condition (benign paroxysmal positional vertigo [26] or urological issues [31]). Five of the studies used 3D models which had been personalised for each patient (e.g., created from the patient’s scans) [27, 29, 32–34], and the remaining five studies used one generic 3D model for all patients [25, 26, 30, 31] and one study had both generic and personalised elements [24].

### Psychological outcomes

The most frequently assessed psychological outcome was anxiety [25–28, 30, 33], but studies also investigated quality of life [24], distress relief [31], and decisional conflict [29]. Types of anxiety measures varied, with three studies using the State-Trait Anxiety Inventory (STAI) [25, 30, 33], two using both a Visual Analogue Scale (VAS) [25, 30], three adopting a single question they developed [26–28] Two studies found that education using 3D models significantly reduced anxiety among patients [26, 27], two studies found there to be mixed results [25, 30], one study found that the intervention did not make any significant difference to anxiety levels compared to standard information using 2D charts [28], and one case study found that anxiety increased following the personalised VR intervention [33]. It should be noted that two studies that found reduction in anxiety used one item measures the authors developed: Fontenot et al.’s [26] study measured anxiety in relation to knowledge about the study specifically and McDonald and Shirk [27] measured reduction in anxiety about condition or treatment with no pre-intervention measure and therefore it is hard to compare these findings with others. In addition, the latter study compared the use of personalised digital 3D models in patients undergoing renal mass treatment either through telehealth or in-person appointments with no control group.

Interestingly, for both studies which used the STAI and VAS [25, 30], the VAS but not the STAI showed anxiety to be reduced following education with generic 3D models in Grab et al.’s study [30] and near statistically significant decrease in Biro et al.’s study [25] compared to the comparison group (education-based only). It should be noted that Biro et al.’s study was powered to detect differences in STAI anxiety and the study did not reach its recruitment goal (still 91.1% recruitment rate) and baseline anxiety was not as high as anticipated. Also, Grab et al.’s study comparing printed 3D (generic) and VR models found anxiety to be significantly reduced for patients in the VR models group but not for those who were in the printed 3D models group and only significantly when anxiety was measured by VAS (the decrease in STAI was not significant) [30].

Benson et al. [24] assessed the effect of using 3D models on quality of life (measured with the Functional Assessment of Cancer Therapy - Lung (FACT-L)) [24] and found no significant change in pre-post intervention and compared to standard education. It should be noted that this study used a personalised multimedia education platform that included digital interactive 3D model of lungs (personalised to each patient’s cancer with surgeon annotations) as well as video tutorials on lung resections. Therefore, the findings cannot be attributed to the impact of 3D digital model alone. The study assessing decisional conflict (using the Decisional Conflict Scale (DCS)) found this variable to be significantly reduced following the intervention (however, there was no control group as it was a feasibility study) [29].

In sum, three quantitative studies using printed 3D models reported positive results [26, 29, 31]. and two reported mixed results [25, 30] and one reported no difference between intervention and standard information group with only post-intervention measure [28]. Of the three studies using digital models, only one showed reduction in anxiety [27], one showed no difference in quality of life between multimedia intervention and standard preoperative education [24] and one that used VR in a case study of one patient showed increase in anxiety [33]. Of the two quantitative studies using VR models, only one reported mixed results [30] and one detrimental effect on anxiety [33]. For those studies using personalised 3D models two reported a positive result [27, 29], one reported negative results [33]. And for the studies using generic 3D models, two positive results were reported [26, 31], with the remainder showing mixed results [25, 30] and one showing no difference [28]. One study that had both generic and personalised elements had mixed results on quality of life [24].

Anxiety and distress relief were also two key psychological constructs identified from the qualitative data extracted from the two interview studies [32, 34]. The constructs identified were discussed as having the potential to be impacted either positively or negatively. For example, Phelps et al. [32] reported in a study with patients with hip injury that some found that viewing the digital personalised 3D models was empowering, with researchers reporting that:

*Three-dimensional images could empower patients*,* enabling them to make sense of their hip condition*,* make a decision about treatment*,* and move forward. (p. 338)*

In this study, one participant commented about the impact of the 3D model on their anxiety in a positive way:

*I am a bit more relaxed about treatment because being able to see it I understand it better so I am not maybe as anxious about it (p. 340*, [34]*).*

Phelps et al. [32] commented that the personalised models:

…*could evoke a sense of relief and reassurance for patients and could reduce their anxiety about treatment (p. 340).*

On the other hand, researchers also found that it could have the opposite effect:

*Images could potentially be unhelpful for some patients*,* causing distress and disempowering them (p. 338)*

This was reported especially when the image did not contain any known abnormality and there was nothing clear to see in the image.

Phelps et al. [32] reported that:

*One patient felt more anxious after viewing their image and the extent of their abnormality [.] Another patient hypothesized that their image may have made them more anxious should their condition have been more serious (p. 340)*

van de Belt et al. [34] also reported mixed results among glioma patient when it comes to psychological impact of seeing 3D printed personalised representations of their brain tumour. The authors reported that the 3D model helped the participants understand their condition, treatment options and risks better with clearer understanding of the size and the location of the tumour than the 2D images. There was also improved communication reported with their neurosurgeon enabling of asking more questions as well as improved communication with friends and family by taking the model home. It was also reported that the models make it very realistic and therefore uncomfortable viewing. Anger and fear were also mentioned as negative effects of 3D printed models.

## Discussion

This scoping review identified evidence for the use of 3D models in clinical consultations with a variety of patient populations, the majority of which were patients about to undergo surgery. Several psychological outcomes were assessed, the most common of which was anxiety which produced mixed results. Evidence for the effect of using 3D models on other psychological outcomes was limited, but quality of life, distress, and decisional conflict had also been explored in the literature, again with mixed results. As the evidence on different psychological variables was limited and the psychometric measures used lacked consistency with different distress measures showing different profiles, even for the larger subset of studies which measured anxiety, we were not able to make firm conclusions about the potential for 3D models to improve psychological outcomes.

Printed 3D models were used in most studies. In some studies, these were individualised models, and in other studies a generic model was used for all patients. Printed models may allow for a better understanding of relevant anatomy and physiology compared with 3D images, as giving the ability to patients to manually interact with models can facilitate learning about the clinical anatomy of their condition [35]. This is shown to be the case for preoperative planning among surgical residents [36] and understanding may be improved among patients as well [11–16]. However, much less is known about how increased patient understanding impacts the patient’s emotional responses and subsequent thought processes, although the findings from this review suggest that anxiety, distress, and decisional conflict may be reduced. Psychological distress and anxiety are found to be associated with extended hospital stays, longer recovery times, and poorer functional outcomes following surgery [37, 38], whilst decisional conflict is indicative of ineffective shared decision-making between patients and clinicians [39]. It is, therefore, clear that poor psychological outcomes following medical consultations have significant implications and there is a need to further explore the psychological effects of using 3D technologies with patients.

The most explored psychological outcome in the literature reviewed was anxiety. The prevalence of anxiety before surgery can be as much as 97% amongst patients [40]. It can have severe repercussions that can be both physical (higher postoperative pain and worsened physical function) and psychological (increased fatigue and depression, and lower quality of life) [41, 42]. Heightened anxiety before surgery can also lead to prolonged hospital stays and higher analgesic consumption [40]. In addition, patient anxiety is not only experienced in consultations occurring prior to a surgery, but also in consultations regarding diagnosis and illness management. We found research showing that 3D models have been used in both types of consultation.

An important influencing factor in the effectiveness of using 3D models to reduce patient anxiety may be the source of the anxiety. For patients facing surgery, this anxiety may be focused on the surgical process or postsurgical experience and outcomes [43], whereas for those receiving information about a diagnosis and prognosis of a condition, anxiety may be associated with feeling able to self-manage, or with making important treatment decisions [44, 45]. Awareness of the source of anxiety can be used to inform the appropriateness of using 3D models. For example, to address anxiety associated with self-management and decision-making, consultations with 3D models can identify issues raising anxiety and lead to appropriate psychological interventions addressing relevant health beliefs and health behaviours can be put in place [46, 47]. Conversely, patients with a high level of surgery-related anxiety facing an upcoming procedure may not benefit from exposure to 3D models or may wish to indicate their preference for receiving other forms of visual information (e.g., medical drawings or leaflets). Some of the studies we identified demonstrated that using 3D models can have an adverse effect by increasing rather than reducing anxiety, as the reality of the medical condition becomes “more real and frightening” [32]. 3D models can be effective in reducing patient anxiety, but more exploration is needed into which patient groups, which types of consultations they are most appropriate for and when and how they can be incorporated into a consultation. It should also be noted that the difference in tools used to measure anxiety may also be an important factor in the findings reported here.

### Implications

This scoping review furthers our understanding of the utility of 3D models in clinical consultations in that they not only have the potential to enhance patient understanding and satisfaction but can also be used to decrease patient anxiety and distress and enable patients to make more informed choices about their treatment. The identified studies used mostly quantitative methodologies and future researchers need to carefully consider which distress measure they use. Further qualitative exploration would allow for a better understanding of the patient experience with 3D models and shed light on the barriers and facilitators to using 3D models effectively in patient education and communication to have optimal outcomes for understanding and changing affective responses. Consideration must also be given to which patient populations would most benefit from 3D models. Much of the research in this space has been conducted in the field of urology [11–16], with the use of 3D technology showing potential with this patient population. Learning more about the patient experience will be an essential next step towards integrating this technology into clinical practice.

When considering the effect of medical consultations on cognitive and affective psychological outcomes, another factor which must be considered is the role of the healthcare professional. The communication style of the healthcare professional can greatly influence patient outcomes. Communication that is warm, empathic, and supportive can significantly reduce patient anxiety [48] and research shows that brief and basic communication skills training can be effective at improving the performance of healthcare professionals [49]. In future research involving intervention with 3D models, clinician knowledge and use of empathic communication strategies should be considered, to investigate the mediational influence of communication style on the effect of educational tools such as 3D models on patient outcomes.

### Limitations

We were not able to produce a comprehensive overview of the cognitive and affective psychological outcomes of using 3D models in medical consultations due to the lack of evidence available, as well as the lack of studies using standardised outcome measurement, despite the employment of a rigorous, systematic approach to identifying relevant research [21]. We acknowledge that different keywords like ‘modeling’ as well as ‘modelling’ and ‘additive manufacturing’ as well as ‘3D’ could have influenced the articles we found. Psychological outcomes other than anxiety were explored but the literature was sparse. Furthermore, the use of 3D models has been trialled in consultations with different purposes, including diagnosis of a condition, discussion of treatment options and preparation for a surgery. Clarification is needed on the types of patients and types of consultations for which 3D models would be most appropriate.

## Conclusion

This scoping review has demonstrated that 3D models can be an effective tool in patient education, particularly when printed models are used. As well as improving patient understanding and satisfaction, they can be used to reduce patient anxiety. Further exploration is needed to learn more about the patient perspective on 3D models (e.g., individualised versus generic), which can be achieved with qualitative methodologies, and through investigation of specific cognitive and affective processes underpinning optimal patient outcomes. The research in this area has been conducted largely in isolation across a variety of medical fields, with a lack of standardised measurement and inconsistency across outcomes measured. Psychological outcomes may differ depending on the health condition being managed and the goals of the consultation, whether that is to support illness management or facilitate decision-making about treatments. Ultimately, medical consultations should prioritise the principles of shared decision-making; (1) working together, (2) discussing options, and (3) making informed choices, with patient wellbeing at the forefront [50]. Preliminary findings show that 3D technology has the potential to facilitate shared decision-making and significantly improve consultation outcomes and warrants further study.

## Data Availability

No datasets were generated or analysed during the current study.

## References

[1] Lim L, Chow P, Wong C-Y, Chung A, Chan Y-H, Wong W-K, et al. Doctor–patient communication, knowledge, and question prompt lists in reducing preoperative anxiety – A randomized control study. Asian J Surg. 2011;34:175–80. 22464834 10.1016/j.asjsur.2011.11.002

[2] Liénard A, Merckaert I, Libert Y, Delvaux N, Marchal S, Boniver J, et al. Factors that influence cancer patients’ anxiety following a medical consultation: impact of a communication skills training programme for physicians. Ann Oncol. 2006;17:1450–8. 16801333 10.1093/annonc/mdl142

[3] Kruzik N. Benefits of Preoperative Education for Adult Elective Surgery Patients. AORN J. 2009;90:381–7. 19735761 10.1016/j.aorn.2009.06.022

[4] Onyemachi J, Pinto-Cuberos J, Miller D, Wagner RF, Winsett F. 3D models for mohs micrographic surgery: a review on its use in patient education. Arch Dermatol Res. 2024;316:470. 39001895 10.1007/s00403-024-03211-w

[5] Burgess LC, Arundel J, Wainwright TW. The effect of preoperative education on psychological, clinical and economic outcomes in elective spinal surgery: a systematic review [Internet]. 2019 [cited 2024 Oct 22]. Available from: https://www.mdpi.com/2227-9032/7/1/48. 10.3390/healthcare7010048 PMC6473918 30901875

[6] Darville-Beneby R, Lomanowska AM, Yu HC, Jobin P, Rosenbloom BN, Gabriel G, et al. The Impact of Preoperative Patient Education on Postoperative Pain, Opioid Use, and Psychological Outcomes: A Narrative Review. Can J Pain. 2023;7:2266751. 38126044 10.1080/24740527.2023.2266751 PMC10732618

[7] Childs S, McVicker Z, Trombetta R, Awad H, Elfar J, Giordano B. Patient-Specific 3-Dimensional Modeling and Its Use for Preoperative Counseling of Patients Undergoing Hip Arthroscopy. Orthop J Sports Med. 2018;6:2325967118794645. 30214907 10.1177/2325967118794645 PMC6134493

[8] Kahsai EA, O’Connor B, Khoo KJ, Ogunleye TD, Telfer S, Hagen MS. Improving Patient Understanding of Femoroacetabular Impingement Syndrome With Three-Dimensional Models. JAAOS Glob Res Rev. 2024;8:e2400116. 10.5435/JAAOSGlobal-D-24-00116 PMC11081616 38722846

[9] Robb HD, Scrimgeour G, Boshier PR, Balyasnikova S, Brown G, Bello F, et al. Current and possible future role of 3D modelling within oesophagogastric surgery: a scoping review protocol. BMJ Open. 2021;11:e045546. 34620652 10.1136/bmjopen-2020-045546 PMC8499311

[10] Habermann AC, Timmerman WR, Cohen SM, Burkhardt BW, Amendola MF. Clinical applications of 3D printing in colorectal surgery: A systematic review. Int J Colorectal Dis. 2024;39:127. 39107626 10.1007/s00384-024-04695-8 PMC11303507

[11] Bernhard J-C, Isotani S, Matsugasumi T, Duddalwar V, Hung AJ, Suer E, et al. Personalized 3D printed model of kidney and tumor anatomy: a useful tool for patient education. World J Urol. 2016;34:337–45. 26162845 10.1007/s00345-015-1632-2 PMC9084471

[12] Choi YH, Lee S-J, Kim HY. Effect of a three-dimensional (3D) printed kidney model on patient understanding of the percutaneous nephrolithotomy procedure: a preliminary study. Urolithiasis. 2022;50:375–80. 35122486 10.1007/s00240-022-01308-3

[13] Esperto F, Prata F, Autrán-Gómez AM, Rivas JG, Socarras M, Marchioni M, et al. New Technologies for Kidney Surgery Planning 3D, Impression, Augmented Reality 3D, Reconstruction: Current Realities and Expectations. Curr Urol Rep. 2021;22:35. 34031768 10.1007/s11934-021-01052-y PMC8143991

[14] Nedbal C, Juliebø-Jones P, Rogers E, N’Dow J, Ribal M, Rassweiler J, et al. Improving patient information and enhanced consent in urology: the impact of simulation and multimedia tools. A systematic literature review from the European Association of Urology Patient Office. Eur Urol [Internet]. 2024 [cited 2024 Oct 22]; Available from: https://www.sciencedirect.com/science/article/pii/S0302283824023054. 10.1016/j.eururo.2024.04.009 38664166

[15] Porpiglia F, Amparore D, Checcucci E, Autorino R, Manfredi M, Iannizzi G, et al. Current Use of Three-dimensional Model Technology in Urology: A Road Map for Personalised Surgical Planning. Eur Urol Focus. 2018;4:652–6. 30293946 10.1016/j.euf.2018.09.012

[16] Wake N, Nussbaum JE, Elias MI, Nikas CV, Bjurlin MA. 3D Printing, Augmented Reality, and Virtual Reality for the Assessment and Management of Kidney and Prostate Cancer: A Systematic Review. Urology. 2020;143:20–32. 32535076 10.1016/j.urology.2020.03.066

[17] Catasta A, Martini C, Mersanne A, Foresti R, Bianchini Massoni C, Freyrie A, et al. Systematic Review on the Use of 3D-Printed Models for Planning, Training and Simulation in Vascular Surgery. Diagnostics. 2024;14:1658. 39125534 10.3390/diagnostics14151658 PMC11312310

[18] de Souza MA, Bento RF, Lopes PT, de Pinto Rangel DM, Formighieri L. Three-dimensional printing in otolaryngology education: a systematic review. Eur Arch Otorhinolaryngol. 2022;279:1709–19. 34533591 10.1007/s00405-021-07088-7

[19] Przedlacka A, Pellino G, Fletcher J, Bello F, Tekkis PP, Kontovounisios C. Current and future role of three-dimensional modelling technology in rectal cancer surgery: A systematic review. World J Gastrointest Surg. 2021;13:1754. 35070078 10.4240/wjgs.v13.i12.1754 PMC8727188

[20] To G, Hawke JA, Larkins K, Burke G, Costello DM, Warrier S, et al. A systematic review of the application of 3D-printed models to colorectal surgical training. Tech Coloproctology. 2023;27:257–70. 10.1007/s10151-023-02757-7 36738361

[21] Levac D, Colquhoun H, O’Brien KK. Scoping studies: advancing the methodology. Implement Sci. 2010;5:69. 20854677 10.1186/1748-5908-5-69 PMC2954944

[22] Arksey H, O’Malley L. Scoping studies: towards a methodological framework. Int J Soc Res Methodol. 2005;8:19–32.

[23] Tricco AC, Lillie E, Zarin W, O’Brien KK, Colquhoun H, Levac D, et al. PRISMA Extension for Scoping Reviews (PRISMA-ScR): Checklist and Explanation. Ann Intern Med. 2018;169:467–73. 30178033 10.7326/M18-0850

[24] Benson J, Bhandari P, Lui N, Berry M, Liou DZ, Shrager J, et al. Use of a Personalized Multimedia Education Platform Improves Preoperative Teaching for Lung Cancer Patients. Semin Thorac Cardiovasc Surg. 2022;34:363–72. 33711462 10.1053/j.semtcvs.2021.03.003

[25] Biro M, Kim I, Huynh A, Fu P, Mann M, Popkin DL. The use of 3-dimensionally printed models to optimize patient education and alleviate perioperative anxiety in Mohs micrographic surgery: A randomized controlled trial. J Am Acad Dermatol. 2019;81:1339–45. 31163232 10.1016/j.jaad.2019.05.085 PMC7031844

[26] Fontenot A, Holmes S, Linquest L, Alexander S, Mankekar G. Helping Patients Understand Their Dizziness: Assessment of a Three-Dimensional Printed Vestibular Model. Indian J Otolaryngol Head Neck Surg. 2023;75:165–9. 37007895 10.1007/s12070-022-03325-5 PMC10050289

[27] McDonald M, Shirk JD. The effect of digital three-dimensional reality models on patient counseling for renal masses. JSLS J Soc Laparosc Robot Surg. 2023;27:e2022.00084. 10.4293/JSLS.2022.00084 PMC9913065 36818764

[28] Sander IM, Liepert TT, Doney EL, Leevy WM, Liepert DR. Patient education for endoscopic sinus surgery: preliminary experience using 3D-printed clinical imaging data. J Funct Biomater [Internet]. 2017;8. Available from: https://www.mdpi.com/2079-4983/8/2/13. 10.3390/jfb8020013 PMC5491994 28387702

[29] Santiago L, Volk RJ, Checka CM, Black D, Lee J, Colen JS, et al. Acceptability of 3D-printed breast models and their impact on the decisional conflict of breast cancer patients: A feasibility study. J Surg Oncol. 2021;123:1206–14. 33577715 10.1002/jso.26420 PMC8011310

[30] Grab M, Hundertmark F, Thierfelder N, Fairchild M, Mela P, Hagl C, et al. New perspectives in patient education for cardiac surgery using 3D-printing and virtual reality. Front Cardiovasc Med [Internet]. 2023;10. Available from: https://www.frontiersin.org/journals/cardiovascular-medicine/articles/; 10.3389/fcvm.2023.1092007. 10.3389/fcvm.2023.1092007 PMC10020687 36937915

[31] Hu W, Song Y, Zhong X, Feng J, Wang P, Huang C. Improving doctor–patient communication: content validity examination of a novel urinary system-simulating physical model. Patient Prefer Adherence. 2016;10:2519–29. 28008237 10.2147/PPA.S123468 PMC5171197

[32] Phelps EE, Wellings R, Kunar M, Hutchinson C, Griffiths F. A qualitative study exploring the experience of viewing three-dimensional medical images during an orthopaedic outpatient consultation from the perspective of patients, health care professionals, and lay representatives. J Eval Clin Pract. 2021;27:333–43. 32488922 10.1111/jep.13417

[33] Rhu J, Lim S, Kang D, Cho J, Lee H, Choi G-S, et al. Virtual reality education program including three-dimensional individualized liver model and education videos: A pilot case report in a patient with hepatocellular carcinoma. ahbps. 2022;26:285–8. 35473767 10.14701/ahbps.21-163 PMC9428435

[34] van de Belt TH, Nijmeijer H, Grim D, Engelen LJLPG, Vreeken R, van Gelder MMHJ, et al. Patient-Specific Actual-Size Three-Dimensional Printed Models for Patient Education in Glioma Treatment: First Experiences. World Neurosurg. 2018;117:e99–105. 29870846 10.1016/j.wneu.2018.05.190

[35] Bücking TM, Hill ER, Robertson JL, Maneas E, Plumb AA, Nikitichev DI. From medical imaging data to 3D printed anatomical models. PLoS ONE. 2017;12:e0178540. 28562693 10.1371/journal.pone.0178540 PMC5451060

[36] Zheng Y, Yu D, Zhao J, Wu Y, Zheng B. 3D Printout Models vs. 3D-Rendered Images: Which Is Better for Preoperative Planning? J Surg Educ. 2016;73:518–23. 26861582 10.1016/j.jsurg.2016.01.003

[37] Hanusch BC, O’Connor DB, Ions P, Scott A, Gregg PJ. Effects of psychological distress and perceptions of illness on recovery from total knee replacement. Bone Jt J. 2014;96–B:210–6. 10.1302/0301-620X.96B2.31136 24493186

[38] Poole L, Kidd T, Leigh E, Ronaldson A, Jahangiri M, Steptoe A. Psychological distress and intensive care unit stay after cardiac surgery: The role of illness concern. Health Psychol. 2015;34:283–7. 25528184 10.1037/hea0000183

[39] Köther AK, Alpers GW, Büdenbender B, Lenhart M, Michel MS, Kriegmair MC. Predicting decisional conflict: Anxiety and depression in shared decision making. Patient Educ Couns. 2021;104:1229–36. 33248869 10.1016/j.pec.2020.10.037

[40] Abate SM, Chekol YA, Basu B. Global prevalence and determinants of preoperative anxiety among surgical patients: A systematic review and meta-analysis. Int J Surg Open. 2020;25:6–16.

[41] Oteri V, Martinelli A, Crivellaro E, Gigli F. The impact of preoperative anxiety on patients undergoing brain surgery: a systematic review. Neurosurg Rev. 2021;44:3047–57. 33608828 10.1007/s10143-021-01498-1 PMC8593022

[42] Villa G, Lanini I, Amass T, Bocciero V, Scirè Calabrisotto C, Chelazzi C, et al. Effects of psychological interventions on anxiety and pain in patients undergoing major elective abdominal surgery: a systematic review. Perioper Med. 2020;9:38. 10.1186/s13741-020-00169-x PMC7722323 33292558

[43] Abelson JS, Chait A, Shen MJ, Charlson M, Dickerman A, Yeo HL. Sources of distress among patients undergoing surgery for colorectal cancer: a qualitative study. J Surg Res. 2018;226:140–9. 29661279 10.1016/j.jss.2018.01.017

[44] Kidd T, Carey N, Mold F, Westwood S, Miklaucich M, Konstantara E, et al. A systematic review of the effectiveness of self-management interventions in people with multiple sclerosis at improving depression, anxiety and quality of life. PLoS ONE. 2017;12:e0185931. 29020113 10.1371/journal.pone.0185931 PMC5636105

[45] Matcham F, Rayner L, Hutton J, Monk A, Steel C, Hotopf M. Self-help interventions for symptoms of depression, anxiety and psychological distress in patients with physical illnesses: A systematic review and meta-analysis. Clin Psychol Rev. 2014;34:141–57. 24508685 10.1016/j.cpr.2014.01.005

[46] Jäger M, Zangger G, Bricca A, Dideriksen M, Smith SM, Midtgaard J, et al. Mapping interventional components and behavior change techniques used to promote self-management in people with multimorbidity: a scoping review. Health Psychol Rev. 2024;18:165–88. 36811829 10.1080/17437199.2023.2182813 PMC7615688

[47] Rask MT, Frostholm L, Hansen SH, Petersen MW, Ørnbøl E, Rosendal M. Self-help interventions for persistent physical symptoms: a systematic review of behaviour change components and their potential effects. Health Psychol Rev. 2024;18:75–116. 36651573 10.1080/17437199.2022.2163917

[48] Zwingmann J, Baile WF, Schmier JW, Bernhard J, Keller M. Effects of patient-centered communication on anxiety, negative affect, and trust in the physician in delivering a cancer diagnosis: A randomized, experimental study. Cancer. 2017;123:3167–75. 28378366 10.1002/cncr.30694

[49] Mata ÁN, de Azevedo S, Braga KPM, de Medeiros LP, de Oliveira Segundo GCBS, Bezerra VH. Training in communication skills for self-efficacy of health professionals: a systematic review. Hum Resour Health. 2021;19:30. 33676515 10.1186/s12960-021-00574-3 PMC7937280

[50] Elwyn G, Durand MA, Song J, Aarts J, Barr PJ, Berger Z, et al. A three-talk model for shared decision making: multistage consultation process. BMJ. 2017;359:j4891. 29109079 10.1136/bmj.j4891 PMC5683042

